# A method of gastric conduit elevation via the posterior mediastinal pathway in thoracoscopic subtotal esophagectomy

**DOI:** 10.1186/1477-7819-10-20

**Published:** 2012-01-24

**Authors:** Noriyuki Hirahara, Tetsu Yamamoto, Tsuneo Tanaka

**Affiliations:** 1 Department of Digestive and General Surgery, Shimane University School of Medicine

**Keywords:** Esophagectomy, Gastric conduit elevation, Echo probe cover

## Abstract

**Background:**

Despite efforts to improve surgical techniques, serious complications still sometimes occur. Use of a physiological posterior mediastinal pathway has increased given advances such as automated anastomotic devices and a reduction in the incidence of anastomotic sufficiency. Until now the gastric conduit created has been protected by an echo probe cover and, sown to the ventral side of polyester tape placed through the abdomen to the neck, and then blindly elevated to the neck. We report on a new method of gastric conduit elevation.

**Methods:**

Two 60-cm lengths polyester tape are ligated at both ends to form a loop. An echo probe cover of 10 cm in diameter and 50 cm in length is prepared and the tip cut off, forming a cylinder. The knots in the previously looped polyester tape are inserted into the echo probe cover. The looped polyester tape and echo probe cover is ligated with silk approximately 5 cm in front of the knots on both sides.

After dissection is carried out according to practice, the previously crafted polyester tape is inserted into the chest cavity. One end of polyester tape is fixed to the distal esophageal stump with the clips, with the opposite end fixed to the proximal esophageal stump. The echo probe cover that connects the proximal esophagus and distal esophagus is monitored for the presence of creases along the long axis to ensure there are no twists in the echo probe cover.

We carry out a laparoscopic-assisted perigastric lymph node dissection, make a small skin incision, and guide part of the thoracic esophagus and stomach outside the body.

Either one of the two lengths of polyester tape is connected to the gastric conduit. By pulling up this length of polyester tape from the neck, the gastric conduit can pass through the echo probe cover and be elevated to the neck.

**Results:**

No perioperative complications such as bleeding or difficulty of the gastric conduit elevation were recognized with this method.

**Conclusions:**

This method is considered to serve as a useful technique for gastric conduit elevation.

## Background

Esophageal cancer surgery is invasive and associated with a high incidence of complications. Although improvements have been made in surgical maneuvers and perioperative care, serious complications still occur after esophageal cancer surgery. Recently, thoracoscopic surgery has been gaining popularity as a minimally invasive surgery. As this method of surgery involves only minimal cutting of the intercostals, respiratory function can be retained, which tends to decrease the incidence of pulmonary complications such as pneumonia and atelectasis [1-3]. In addition, the pre-sternal and retro-sternal routes have often been selected for pathway reconstruction in the past due to the possibility of fatal suture failure. Recently, however, the use of a physiological posterior mediastinal pathway has increased due to advances such as automated anastomotic devices and a reduction in the incidence of anastomotic sufficiency [4,5]. At present, a consensus has not been obtained due to the differences between facilities, including preoperative and postoperative management as well as the surgery itself [6-8]. In our department, the standard surgical procedure has been thoracoscopic subtotal esophagectomy from a semi-pronated position and laparoscopic gastric surgery, gastric conduit elevation to the neck via the posterior mediastinal pathway, and anastomosis and reconstruction of the cervical esophagus and gastric conduit at each end. Until now, the created gastric conduit has been protected in an echo probe cover and, after completion of thoracoscopic manipulation, sown to the ventral side of the polyester tape, which has been placed through the abdomen to the neck, and blindly elevated to the neck [9]. However, when this elevation method is used, the gastric conduit adheres to the azygos vein and bronchial arteries and poses a risk of major bleeding. In this paper we report on a safe method of gastric conduit elevation that we have used, which can be performed to reduce the risk of bleeding.

## Methods

The new method was evaluated in a consecutive number of patients. This series was compared to a series using the conventional method.

### Modification of polyester tape and echo probe cover

**Figure 1:**
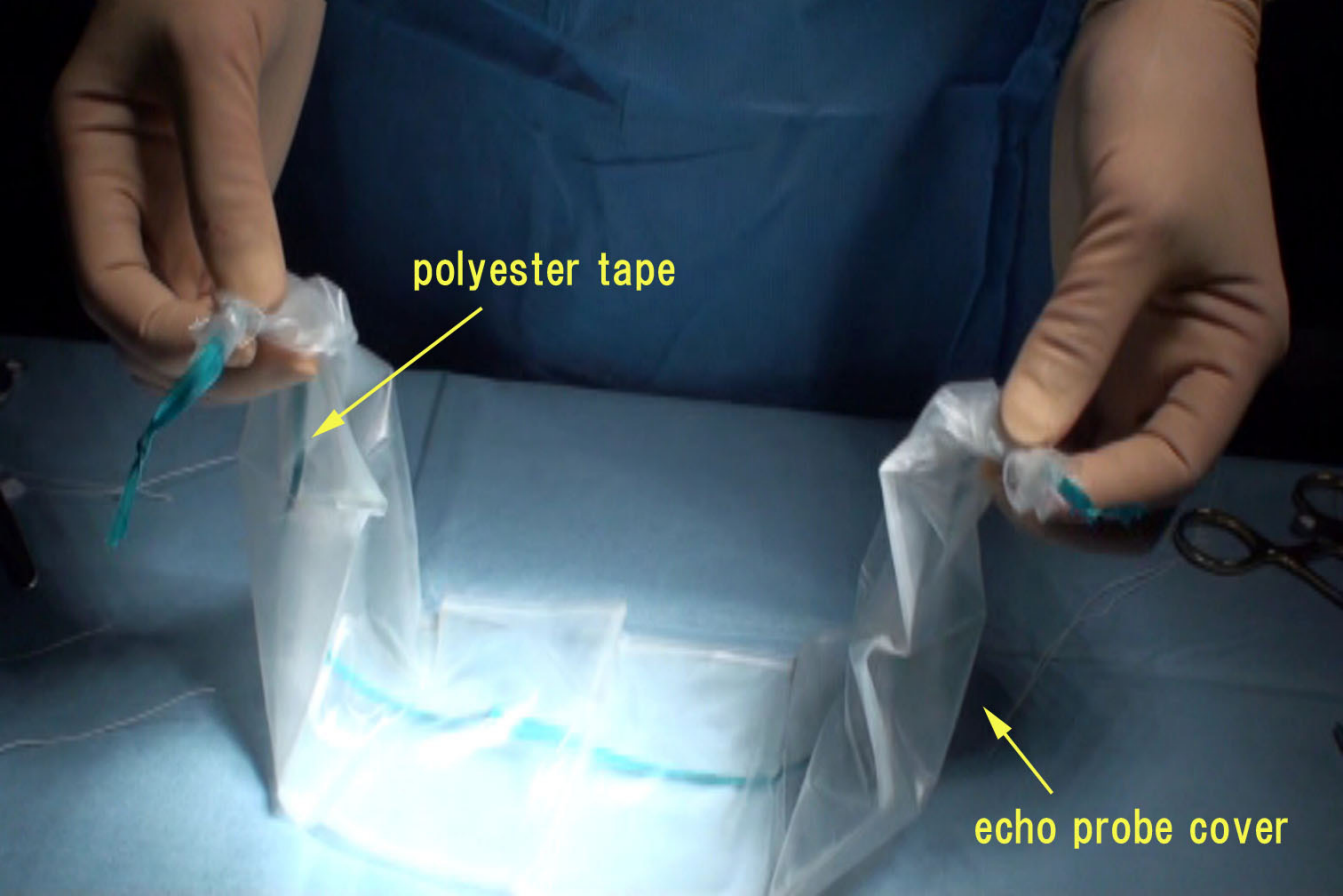
**The looped polyester tape and echo probe cover is ligated with 2-0 silk approximately 5 cm in front of the knots on both sides**.

Two approximately 60-cm-long polyester tapes are prepared and ligated at both ends forming a loop. An echo probe cover of 10 cm in diameter and 50 cm length is prepared, and the closed end of the echo probe cover is cut to make an open-ended tube. The knots in the previously looped polyester tape are inserted into the echo probe cover, so that they reach all the way down. The looped polyester tape and echo probe cover are ligated with 2-0 silk, approximately 5 cm in front of the knots on both sides, and are fixed to avoid misalignment (Figure 1).

### Thoracic manipulation

**Figure 2:**
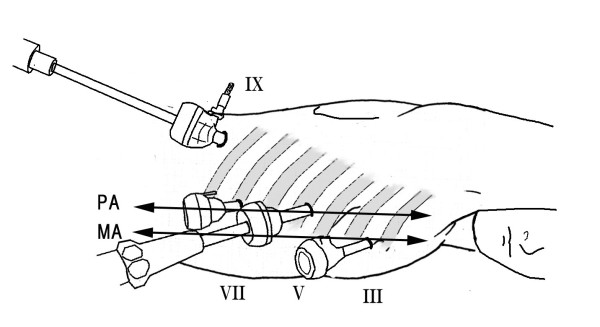
**Position of the patient and sites of the four trocars**. Roman numerals show the rib number. MA midaxillary line, PA posterior axillary line.

A 12-mm blunt trocar is inserted into the fifth intercostal space (ICS) on the posterior axillary line. Another 3 trocars are inserted under thoracoscopic guidance; a 5-mm trocar is inserted in the third ICS behind the midaxillary line, a 5-mm trocar in the seventh ICS behind the posterior axillary line, and a 12-mm trocar in the ninth ICS on the scapular angle line for the thoracoscope (Figure 2). A pneumothorax is made by maintaining a CO_2 _insufflation pressure of 6 mmHg, and esophagectomy is performed in the prone position [10]. Transection of the esophagus at the upper thoracic esophagus is performed using an automatic anastomotic device. After dissection is performed, the previously crafted polyester tape is inserted into the chest cavity from the 12-mm port. Because both ends of the polyester tape are ligated to form loops, the laparoscopic clips hook onto these loops and one end is fixed to the distal esophageal stump with the clips, with the opposite end fixed to the proximal esophageal stump. The echo probe cover that connects the proximal esophagus and distal esophagus is monitored for the presence of creases along the long axis to ensure there are no twists in the echo probe cover. Creases are easier to notice if the echo probe cover is marked along the long axis with a clean pen in advance. Finally, the thoracostomy tube is inserted, and intrathoracic manipulation is completed.

### Abdominal and cervical manipulation

**Figure 3:**
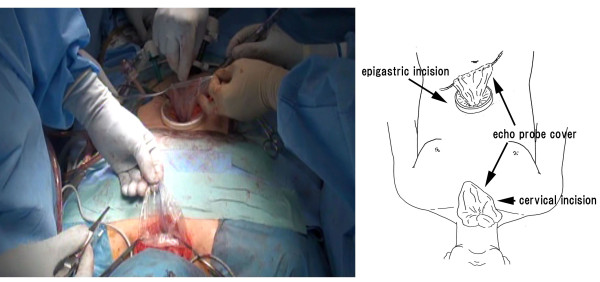
**The polyester tape on the abdominal and cervical side and the echo probe cover are evenly guided outside the body**.

In the supine position, we perform laparoscopic-assisted perigastric lymph node dissection, make a small incision of approximately 5 cm in the epigastric region, and remove a part of the thoracic esophagus and stomach from the body. In this manipulation, the previously crafted polyester tape, which was fixed to the detached distal esophageal stump, is guided outside the body. In addition, in cervical manipulation, the crafted polyester tape, which has been fixed to the esophageal stump, is removed in a manner similar to that used for removing the proximal stump of the thoracic esophagus from the neck. The crafted polyester tape with the attached echo probe cover connects the abdomen and neck through the posterior mediastinal pathway (Figure 3).

### Gastric conduit elevation

**Figure 4:**
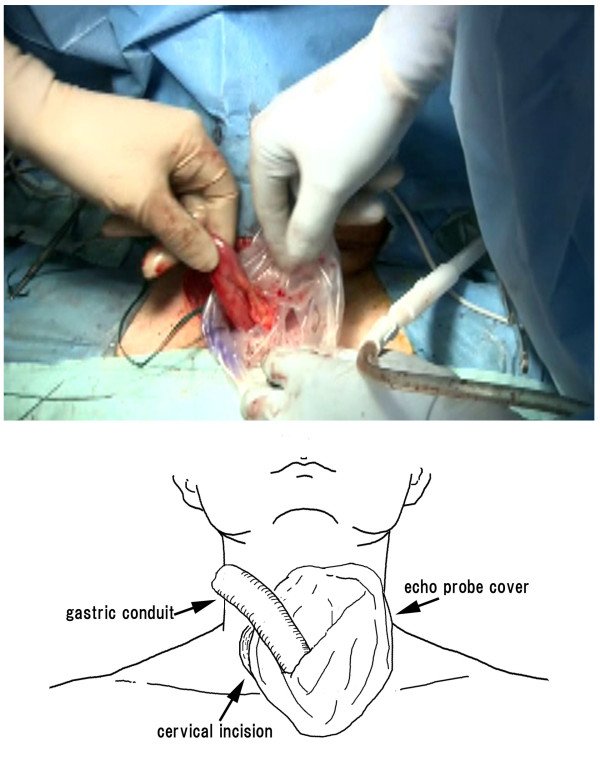
**By pulling up this polyester tape from the neck, the gastric conduit can pass through the echo probe cover and be elevated to the neck**.

The polyester tape on the abdominal and cervical side and the echo probe cover are evenly guided outside the body. The polyester tape and echo probe cover fixing is removed, and the knot in the tape is untied to convert the loop back to two pieces of tapes. One of the two pieces of tape is then connected to the gastric conduit created under direct vision. By pulling up this length of polyester tape from the neck, the gastric conduit can pass through the echo probe cover and be elevated to the neck (Figure 4). If a small amount of saline is injected via a nelaton catheter inserted into the echo probe cover, friction and resistance are further reduced when the gastric conduit passes through the echo probe cover, making elevation easy. We prevent injury to the gastroepiploic vessel, nutrient vessel by reducing resistance. Moreover, manually pushing the gastric tube, from the abdominal cavity, into the echo probe cover in a coordinated manner with the polyester tape-pulling maneuver further facilitates smooth gastric conduit elevation.

When the gastric conduit is elevated to the neck, the echo probe cover is disconnected from the neck and the remaining polyester tape was removed. An additional pneumoperitoneum procedure or maneuver to induce the gastric conduit into the esophageal hiatus is unnecessary as the gastric conduit elevation route could be secured with the echo probe cover.

If the gastric conduit could not be elevated to the neck with the first polyester tape, the second polyester tape will be needed to perform the elevation once again and therefore should only be removed after all manipulations are complete.

### Statistical analysis

Clinical characteristics and surgical results of the two groups were analysed. Statistical analysis was performed with the SPSS 13.0 statistical software. The comparisons among groups were performed by Student's t test and the chi-square test. P values are reported for a two-tailed test with P < 0.05 considered significant.

## Results

Patient demographics and clinical characteristics are listed in Table 1. There was no significant difference between the two groups in age, gender, location of tumor, or staging. All patients of two groups had histological diagnosis of esophageal squamous cell carcinoma.

**Table 1:** Patient demographics and clinical characteristics

	Conventional method (n = 36)	Our new method (n = 8)	P value
Age(years)			
Mean(range)	67(57-79)	68(56-85)	0.787
Gender			
Male/female	30/6	7/1	0.770
Location of main tumor			
Upper	6	1	0.956
Middle	21	5	
Lower	9	2	
Depth of tumor invasion			
T1	7	1	0.245
T2	10	2	
T3	19	5	
T4	0	0	
Lymphnode metastasis			
N0	20	5	0.245
N1	16	3	
TNM stage			
0	0	0	0.220
I	6	1	
II A	14	4	
II B	10	2	
III	6	1	
IV	0	0	

From September 2009 to February 2011, we needed eight ± two minutes with thirty-six patients to elevate the gastric conduit using the conventional method. But from February 2011 to September 2011, we need only two minutes ± two minutes with eight patients using the new method proposed here.

None of these patients showed tumor infiltration into other structures, and curative resection was performed

Postoperative complications developed in 3 of the 8 patients in our new method group (37.5%) and in 14 of the 36 patients of conventional method group (38.9%) (Table 2). There was no significant difference in the incidence of postoperative complications between the two groups. Furthermore no perioperative complications such as bleeding or difficulty of the gastric conduit elevation were recognized with this new method.

**Table 2:** Surgical results of a thoracoscopic esophagectomy

	Conventional method (n = 36)	Our new method (n = 8)	P value
Operation time(min)			
Total	563 ± 57	583 ± 47	0.598
Chest	289 ± 67	313 ± 41	0.498
Gastric conduit elevation	8 ± 2	2 ± 2	0.078
Blood loss(ml)	153 ± 115	137 ± 37	0.633
Mortality	0	0	
Morbidity	14	3	0.941
Respiratory complications	5	1	0.918
Hoarsness	7	2	0.725
Anastomotic leakage	9	1	0.445
Chylothorax	1	0	0.633
Post operative hospital stay (days)	30 ± 41	28 ± 36	0.784

## Discussion

The stomach is the most commonly used organ in esophageal reconstruction after subtotal esophagectomy. When the stomach is judged unsuitable, the small intestine or colon is used via the anterior or posterior sternal route or the posterior mediastinum route [11,12]. Each of these organ reconstruction and route reconstruction has its own advantages and disadvantages and differs according to the medical facility. Recently, increasingly more facilities use the posterior mediastinum route because it offers physiological advantages and short reconstruction distance. The gastric conduit is blindly pulled up to the neck, where an anastomosis connects it to the ventral side of the polyester tape that has been placed through the abdomen to the neck via the posterior mediastinal route, after completing thoracoscopic manipulation [8,9]. However as the gastric conduit is blindly elevated intrathoracically, twists in the gastric tube and damage such as vascular damage to other organs is a risk [13,14]. In the 35 cases of esophageal cancer that we have operated on until now, we performed thoracoscopic subtotal esophagectomy in a semipronated position and reconstructed the posterior mediastinal route by using the stomach. However, the 36^th ^case involved a patient who sustained damage to the right bronchial artery during elevation of the gastric conduit and suffered major bleeding. Fortunately, as the bleeding had stopped when the thoracic cavity was thoracoscopically examined again, we clipped each stump of the bronchial artery, and the surgery was completed without any need for blood transfusion. However, after this experience, we thought it was necessary to construct some sort of device to avoid similar problems in the future.

It is safe to elevate the gastric conduit while observing the thoracic cavity thoracoscopically. However, observing the peritoneal cavity and thoracic cavity at the same time is difficult and positioning is an issue. Using the new method we considered, the echo probe cover can be placed in the posterior mediastinal without twists after completion of thoracic maneuvering. The gastric conduit can then be elevated through the intra echo probe cover. This enables the gastric conduit to be elevated safely to cervical field without contacting other organs. Even if there is a twist in the echo probe cover, resistance can be felt during gastric conduit elevation and the gastric conduit can be elevated together with the echo probe cover, so there is no change from the conventional method in which the gastric conduit is covered and protected by the echo probe cover and no increased risk of damage to other organs. The risk of omental and other damage has become a concern when the gastric conduit passes through the echo probe cover, but resistance is decreased by filling the echo probe cover with saline. We have performed the presented procedure in 8 patients, without injury to the greater omentum. If, however, resistance is felt, it is important to change to a method in which the gastric conduit is elevated to the neck together with the echo probe cover. A dual method of protection such as passing the gastric conduit through the echo probe cover after the gastric conduit has been covered and protected as a method to prevent omental damage has been considered for the future, but has not yet been implemented because omental damage has not yet been experienced.

In maneuvers with laparotomy, the esophageal hiatus can be observed under direct vision and a bent gastric conduit does not interfere with the vision. In laparoscopic surgery, pneumoperitoneum was achieved again during gastric conduit elevation and the gastric conduit was carefully transported to the thoracic cavity using forceps with an emphasis on gastric conduit elevation from the neck, while checking the esophageal hiatus [15,16]. With this manipulation, there were many cases in which the gastric conduit was bent and it was difficult to ensure vision. However, with our method there is no need for pneumoperitoneum to be performed again, and easy elevation is possible by pushing the gastric conduit directly into the echo probe cover by hand though a small epigastric incision. Because many esophageal cancer surgeries take a long time; this causes stress to the operating staff as well as the patient, there is an urgent need to improve surgical procedures and work to reduce their length [17,18]. We wish to continue further development by first accumulating cases with which to evaluate the usefulness of our method.

## Competing interests

The authors declare that they have no competing interests.

## Authors' contributions

NH was the lead author and surgeon for all of the patients. TY contributed patients and information on the patients. TT reviewed paper and technique of surgery. All authors read and approved the final manuscript.
